# TBA for Sensing Toxic Cations: A Critical Analysis of Structural and Electrical Properties

**DOI:** 10.3390/ijms241914492

**Published:** 2023-09-23

**Authors:** Eleonora Alfinito

**Affiliations:** Dipartimento di Matematica e Fisica ‘Ennio De Giorgi’, Università del Salento, I-73100 Lecce, Italy; eleonora.alfinito@unisalento.it

**Keywords:** anti-thrombin aptamer, complex network, electrical response

## Abstract

Food and drinks can be contaminated with pollutants such as lead and strontium, which poses a serious danger to human health. For this reason, a number of effective sensors have been developed for the rapid and highly selective detection of such contaminants. TBA, a well-known aptamer developed to selectively target and thereby inhibit the protein of clinical interest α-thrombin, is receiving increasing attention for sensing applications, particularly for the sensing of different cations. Indeed, TBA, in the presence of these cations, folds into the stable G-quadruplex structure. Furthermore, different cations produce small but significant changes in this structure that result in changes in the electrical responses that TBA can produce. In this article, we give an overview of the expected data regarding the use of TBA in the detection of lead and strontium, calculating the expected electrical response using different measurement techniques. Finally, we conclude that TBA should be able to detect strontium with a sensitivity approximately double that achievable for lead.

## 1. Introduction

Air pollution and food contamination pose a serious risk to human health. The toxicity of some metals is well known, especially arsenic, mercury, lead, and cadmium [1], which have multiple technological applications and therefore are very present in everyday objects and waste. They can come into contact with humans in various forms, through inhalation, in the form of an aerosol, in contact with the skin, and, finally, via ingestion through food. In particular, lead is present both in the air and in the water and tends to accumulate in the surface soil, compromising the development of crops [2]. It produces hematological and neurological diseases, particularly in children, even at low concentrations, and can induce several different diseases, anemia, cancer [1], and renal failure [3], to name the most common.

Some other metals, however, such as iron or calcium and even strontium, are believed to be useful for various physiological activities and for general human well-being.

In particular, strontium, an alkaline metal considered non-toxic, finds various applications in medicine; it is quite similar to calcium and manganese with regard to biological interactions, even if often, in humans, the response to strontium is weaker than that of calcium. This metal is then used like calcium for healing teeth, bones, and tissue [4].

However, recently, there have been some warnings about possible effects on the heart in patients treated with strontium [5]. Furthermore, potential risks to human health have been highlighted by a study conducted on strontium-contaminated water, which poses a serious vulnerability because this metal can be present in high quantities in volcanic rocks [6] and therefore in drinking water.

Several types of biosensors have been developed to detect lead, some of which are based on aptamers [7], i.e., small strands of DNA and RNA synthesized to bind a specific target with high affinity [8].

Different techniques have been used to monitor the presence of pollutant ions in different substrates. Fluorescence was used to detect Pb^2+^ in tea samples [9], while an electrochemical measurement confirmed the selectivity of aptamers for lead and mercury [3]. In [10], an FET-based aptasensor was developed for sensing lead in blood. This article investigates the use of the 8-17DNAzyme aptamer, showing a difference of about nine orders of magnitude in the detection of Pb^2+^ compared to other cations such as Na^+^, K^+^, Mn^2+^, and Ca^2+^. This high selectivity allows it to be used in an array of complex samples. 

Aptamers are small biomolecules (less than 50 kDa) with a much more flexible structure than normal proteins. Revealing their 3D structure is still a challenge, both using physical–chemical methods such as NMR and X-ray crystallography [11,12,13,14], and in silico methods [15,16], recently also based on AI [17].

In recent years, much attention has been paid to the aptamer called TBA (5′-GGTTGGTGTGGTTGG-3′) [18], a guanine-rich oligomer that has a high affinity for a protein of great clinical interest, α-thrombin. Specifically, this aptamer is able to inhibit protein activity, reducing the formation of clots.

Regarding its 3D structure, most of the information comes from NMR analyses. Researchers detected a G-quadruplex arrangement, which is a peculiar conformation in G-rich oligomers: two G-quartets are linked by two TT loops at one end and a TGT loop on the other end [15]. The G-quadruplex configuration has always been resolved in the presence of monovalent or divalent cations [9,18,19,20,21,22,23,24], which would seem to play a fundamental role in its stabilization.

The structure of TBA has been resolved through X-ray crystallography when bound to thrombin [23] with which it forms a stable complex. It has been shown that the presence of two different cations, K^+^ and Na^+^, causes tiny but sensitive changes in the structure, inducing a different ability to inhibit the target protein. Only recently has it been possible to resolve TBA in its free state in the presence of Pb ^2+^ through X-ray spectroscopy [9].

In each case studied, the specific G-quadruplex shape assumed by TBA depends on the size and charge of the cations, for example, K^+^ is larger than Na^+^ and consequently the aptamer structure is more dilated [23]. Furthermore, it has been demonstrated that the G-quadruplex structure formed in the presence of Na^+^ is less stable than the structure obtained in the presence of K^+^ [25].

The binding of 15-mer and 12-mer oligomers with Mn^2+^ has been explored in [19]; both aptamers assume a stable structure in the presence of the cation; and, in particular, while the 15-mer, TBA, acquires the characteristic G-quadruplex structure, the 12-mer, non-TBA, takes the form of a basket.

The first topological data on TBA stabilized in the presence of Sr^2+^ were NMR data [22]; in that paper, a mean distance greater than that of the TBA-K^+^ complex was detected, also suggesting a different binding site.

In conclusion, the literature suggests that, in general, different cations are useful for stabilizing TBA in the G-quadruplex structure, producing small differences. It is remarkable to observe that the structural differences, although tiny, produce significant variations in the functioning of the TBA [23,25]. Furthermore, as suggested in [10,26], aptamers can be used, after proper calibration, as a very fine sensor of various toxic and non-toxic cations.

In this article, we carry out a theoretical investigation regarding the presence of different cations in determining the final structure of the TBA. This allows us to predict the electrical response of this aptamer when used to design a sensor for the detection of toxic or potentially toxic cations.

## 2. Results and Discussion

We analyze some topological and electrical characteristics of the G-quadruplex structure of TBA in the presence of different cations. To this end, the theoretical methods described in Section 3 are adopted. The aptamer is described in terms of an equivalent network whose topology and response to electrical stimuli give a picture of what takes place in real macromolecules.

Below, we report the data coming from both X-ray crystallography and NMR analysis. Specifically, since NMR analysis produces several conformers, they are all examined and minimal differences are found between them. Therefore, only the data relating to the best representative conformer, as given in the Protein Data Bank (PDB) [11], are reported.

### 2.1. Structural Analysis

**Figure 1:**
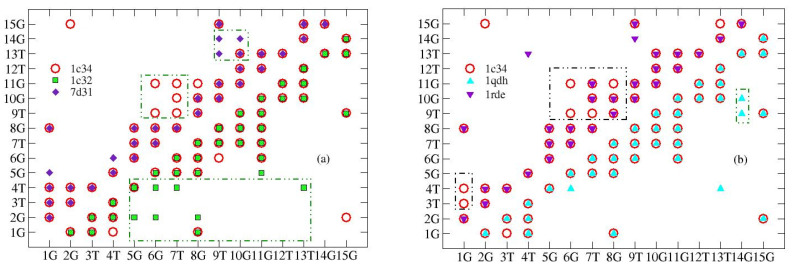
Contact maps of the TBA G-quadruplex structure in the presence of different cations. The reference structure, resolved in the presence of potassium (empty circles), is compared with structures resolved in the presence of (**a**) lead (full diamond), above the diagonal, and potassium with stoichiometry 2:1 (full squares), below the diagonal; and (**b**) manganese (triangle up), below the diagonal, and strontium (triangle down), above the diagonal. Boxes highlight the aptamer regions in which major differences appear.

As detailed in Section 3.1, the macromolecule is described in terms of an impedance network and the contact maps allow its connections to be represented; each pair of connected nodes (*l*,*m*) of the network is a symbol in the map and the maps are symmetric. The complete list of analyzed structures is reported in the Table in Section 3.2. The reference structure, 1c34, solved in the presence of K^+^, is graphically represented both above and below the diagonal, while the other structures are drawn above and below the diagonal (see Figure 1).

Pb^2+^ and Sr^2+^ (PDB entries: 7d31 and 1rde, respectively) modify the structure more than K^+^. The differences are localized in the central part of the aptamer (6G-8G): here, some contacts are missing, indicating a dilatation of the structure. Furthermore, Pb^2+^ adds some contacts between 9T, 10G, and the aptamer tip, suggesting a folding in this region. The structure obtained using potassium with 2:1 stoichiometry (file PDB: 1c32) produces significant differences with respect to the 1:1 stoichiometry; in fact, it affects the whole aptamer, producing many more bonds between 2G and 4T and the other nucleobases. Finally, the structure obtained in the presence of Mn^2+^ (PDB entry: 1qdh) does not present substantial differences with respect to the reference structure.

**Figure 2:**
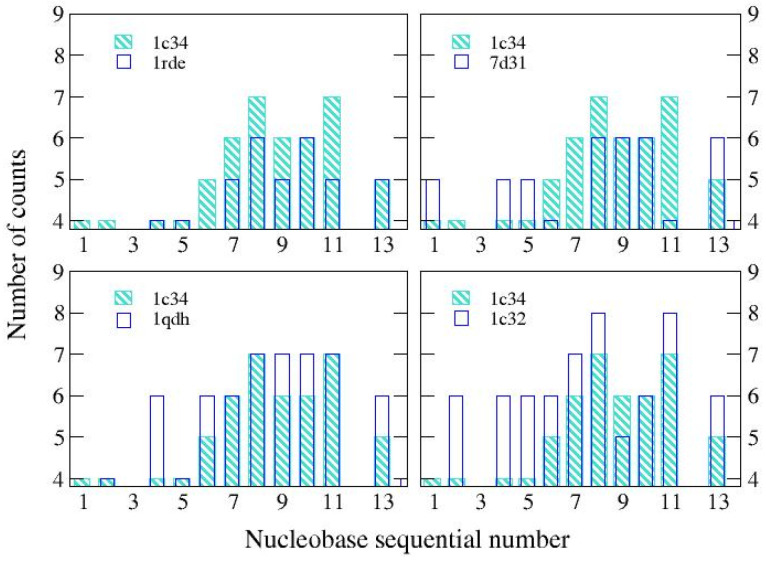
Distribution of links for the analyzed structures. The reference structure, resolved in the presence of K^+^ 1:1 (1c34), is compared with those obtained in the presence of Sr^2+^ (1rde), Pb^2+^ (7d31), Mn^2+^ (1qdh), and K^+^, 2:1 (1c32).

Looking globally at the link distribution (Figure 2), we can see that for both the reference structure and 1qdh (Mn^2+^), most of the links are found around the central nucleobases of the aptamer (6G-11G). Both 1rde (Sr ^2+^) and 7d31 (Pb ^2+^) show a reduction in the bonds in this region, without significant variations compared to the other nucleobases; therefore, the global effect is an expansion. Finally, the structure with potassium (2:1), 1c32, shows a greater number of bonds and a more uniform distribution; this should be related to the presence of the second cation located in a different binding site. In this way, the structure is globally more connected.

### 2.2. Resistance Analysis (Low-Bias Regime)

**Figure 3:**
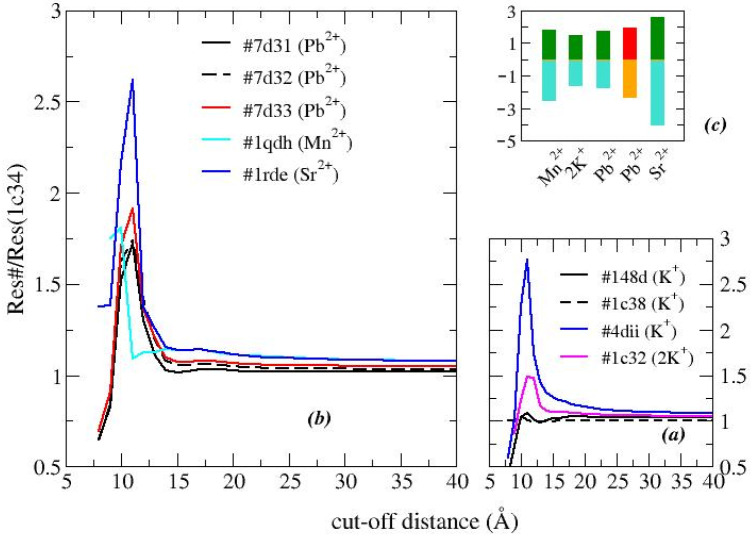
Relative resistances of TBA G-quadruplex structures in the presence of different cations. (**a**) TBA in the presence of potassium with stoichiometry 1:1 (PDB entries: 148d, 1c38, 4dii) and 2:1 (PDB entry: 1c32); (**b**) TBA in the presence of lead (PDB entries: 7d31 and 7d32; mutant G8C, PDB entry 7d33), strontium (PDB entry: 1rde), and manganese (PDB entry: 1qdh); and (**c**) bar-plot of the maximum value of the relative resistance (top) and of the area under the resistance curve (bottom). Red/orange bars refer to the mutant G8C. Color online.

As detailed in Section 3, the effective resistance of various G-quadruplex structures is calculated in the low-voltage regime (less than 100 mV), over a wide range of cut-off values (*D*), i.e., the parameter that determines the degree of connection of the network. The analysis of the free aptamers and also of a TBA structure complexed with thrombin (although lacking the protein) is shown in Figure 3. The resistance of the network decreases as the value of *D* increases [27,28]; in fact, a more connected network is equivalent to having more channels for charge transfer, thus increasing the current flow. This is true for all types of networks, so it does not give much information.

However, by comparing the resistances of two different structures, it is possible to collect some interesting data; in particular, it is possible to detect whether the differences concern the entire structure or if they are localized [27,28].

Analyzing the effects of the different cations, it is observed that the structure stabilized in the presence potassium ions, with stoichiometry 1:1 (PDB entry 1c34), produces the least resistance; therefore, it is chosen as the reference structure. 

We observe that for the analyzed structures, as *D* increases, the resistances converge toward similar values, which means that the differences between the structures are limited to small regions. This confirms what was previously observed (Figure 1 and Figure 2), namely that the binding sites of the various cations are very close to each other and the deformations of the structure are concentrated there. 

An exception is the structure formed in the presence of two potassium ions (1c32). In fact, the second potassium ion appears to produce a folding in part of the structure rather than an expansion (Figure 2). On the other hand, its resistance is greater than that of the reference structure (single potassium ion), which indicates that this modification does not concern the region most crossed by the current.

As the first test, we compare the structural data of TBA stabilized in the presence of the same cation but classified differently in the PDB: 148d, 1c34, and 1c38 for K^+^ (1:1); 1c32 and 1c35 for K^+^ (2:1); and 7d31, 7d32, and 7d33 (see Figure 3a,b). We observe that their answers are almost superimposable. A slight increase in resistance is observed for the Pb^2+^-G8C mutant (cysteine replaces guanine 8). 

The binding with thrombin further deforms the structure obtained in the presence of K^+^ (PDB entry: 4dii), here deprived of the protein. The relative resistance curve is similar to that given by TBA-free although with a much higher maximum value. This result confirms that the docking with the protein further deforms the aptamer, although the region of deformation remains confined to that of the free aptamer. The value of *D* that maximizes the differences is 11 Å.

Ultimately, ions other than potassium produce larger differences than the reference structure. In particular, in the presence of Pb^2+^, and even more of Sr^2+^, the relative resistance is higher than that of K^+^. Manganese appears to produce a similar result even when maximized over *D* = 10 Å.

Further information is given by considering the entire region in which the difference occurs, so we can compare the different areas under the curve. The data are reported in Figure 3c and confirm that Sr^2+^ produces the greatest deformation not only locally.

### 2.3. Impedance Analysis (Low Bias Regime)

**Figure 4:**
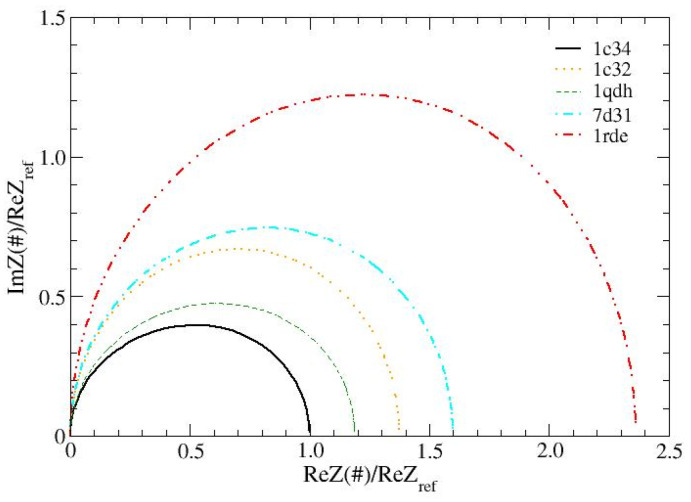
Impedance spectra calculated for the analyzed TBA structures. All curves have been normalized to the maximum impedance of the reference structure, 1c34 (K^+^, 1:1). *D* value is 10.5 Å, frequency range is 0.1–10^6^ Hz.

The global impedance of the aptamer is calculated as described in Section 3. This quantity, measured over a wide range of frequencies, is the typical output of an investigation conducted using electrochemical impedance spectroscopy (EIS) [27,28,29]. It is reported in the so-called Nyquist graphs, which, in our case, represent calculated and not measured data. The Nyquist graphs, normalized to the maximum impedance of the reference structure, are shown in Figure 4. These data strongly suggest that the measurements carried out using EIS are able to resolve, after an appropriate calibration, the presence of different cations. In fact, each of the cations considered produces an almost semicircular pattern and the structures chelated with Pb^2+^ and Sr^2+^ have the largest radius. Furthermore, as a distinctive trait, actual departures from the semicircular shape are mainly observed for the reference structure 1c34 and 1qdh and become even less marked for the other structures. This behavior signals the presence of different characteristic times and thus appears to be due to the different organization of the structures that, in 1c34 and 1qdh, are very connected in the central part (hub), while the others have much more uniformly distributed links (see Figure 2). 

### 2.4. Conductance Analysis (Intermediate and High Bias) 

Low- and intermediate-bias current measurements are increasingly used in biosensing applications involving aptamers [30] or DNA strands [31].

Atomic force microscopy (AFM) is a widely used technique in aptamer analysis, mainly for topography [25], to measure target affinity [32] and also to modulate the strength of binding [33], while conductive-AFM for aptamer applications is still in its infancy [34]. These types of techniques allow for the exploration of a large voltage range that can detect super-linear responses. 

For small applied biases, our model describes a linear electrical response, in agreement with much experimental data [35,36,37]. On the other hand, the deviation from linearity is expected as the voltage intensity increases and it is interesting to estimate the voltage value that determines the onset of the super-linear response. The conductance, which is constant in the linear (Ohmic) regime, is a useful tool for estimating this value [35,36,37].

**Figure 5:**
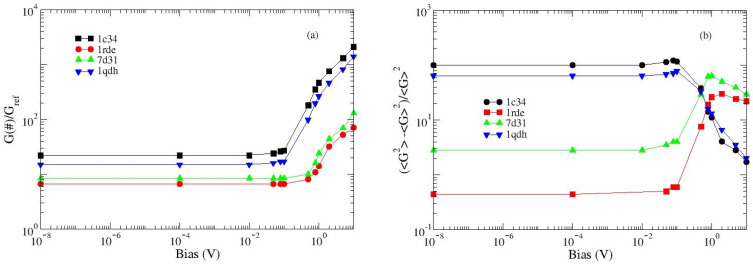
Calculated conductance and conductance variance for the analyzed structures. (**a**) Normalized conductance; the reference value is the conductance of 1c34 (K^+^, 1:1), calculated at the lowest bias. (**b**) Conductance variance. Symbols are calculated values and lines are eye guides.

The conductance is analyzed in the bias range (0.1 mV–10 V): it remains constant up to about 0.1 V, for 1c34 (K^+^, 1:1) and 1qdh (Mn ^2+^), and up to about 0.8 V, for 1rde (Sr^2+^) and 7d31 (Pb^2+^); for higher voltage values, a deviation from linearity appears. The data reported in Figure 5a concern the mean values calculated over 10 realizations, each comprising 4 × 10^5^ iterations. Some additional information comes from the analysis of the conductance fluctuations (Figure 5b). At low bias, they are usually quite high, because the two tunneling regimes described in Equation (4) coexist [35,36,37]. On the other hand, when the voltage increases, only one of them survives and the fluctuations are reduced. The beginning of the non-linear regime coincides with the maximum of the fluctuations.

The response of the structures resolved in the presence of lead and strontium is quite different from those of the other two analyzed; this behavior, useful for discriminating between different structures, however, deserves further investigation.

## 3. Materials and Methods

### 3.1. Methods

The analyses were conducted in the context of proteotronics [35,37], a method of theoretical investigation that analyzes the topological properties of biological macromolecules, associating them with a network of electrical elements.

The structural and electrical characteristics of the network depend on the macromolecule topology and its chemical composition. The deformation of structure produces changes in the electrical response of the network. Briefly, the oligomer is mapped into (i) a set of nodes, each representing a single nucleobase, and (ii) a set of links that connect a couple of nodes when they are closer together than an assigned cut-off distance, *D*. Increasing the value of *D* means increasing the number of nearest neighbors and, in turn, making the network more connected. Too small values of *D* produce too simple networks (almost 1*D*), which do not allow the structure of the macromolecule to be appreciated, and vice versa, too large values of *D* produce a completely connected network, which is useless for appreciating the specific topology and the electrical characteristics of the aptamer. The best choice is in the range of 5–12 Å [35,36,37]. This topological network, far from being regular, has a small-world structure [27]. The main information relating to the results described in Section 2 is presented below.

- Contact maps

The structural properties of the network mirror those of the macromolecule. In particular, for an assigned value of *D*, it is represented by an N×N Boolean (symmetric) matrix of elements 1/0, where N is the number of nucleobases. The value 1 is attributed to a couple of connected nodes; otherwise, it is zero. The graphical representation of this matrix is shown in the contact map in Section 2.1.

- Resistance and conductance

The electrical response of macromolecules when brought into contact with an appropriate bias has been analyzed in previous investigations [27,35,36,37], which produce results in good agreement with the experiments. The technique consists in associating each link of the network with a circuit element chosen to describe the main characteristics of living matter: charge transfer (high resistance) and charge separation (polarization).

Specifically, each link connecting a pair of nodes (*l*,*m*) is associated with:

(i) An elementary resistance to represent the charge transfer:

(1) $$R_{l,m}=\rho_{l,m}\left[\frac{4d_{l,m}}{\pi \left(D^{2}-{d_{l,m}}^{2}\right)}\right]$$

where $\rho_{l,m}$ is the resistivity of the link, calculated as described in [28], and $d_{l,m}$ is the Euclidean distance between the nodes (*l*,*m*);

(ii) An elementary capacitance to represent polarization:

(2) $$C_{l,m}=\varepsilon_{l,m}\left[\frac{\pi \left(D^{2}-{d_{l,m}}^{2}\right)}{4{\;d}_{l,m}}\right]$$

where $\varepsilon_{l,m}$ is the dielectric constant of the nucleobase couple [28]. 

The elements described by Equations (1) and (2) are connected in parallel and the global impedance is calculated; in this way, it depends on the specific topology of the macromolecule and constitutes a probe sensitive to the change in the structure. Finally, the global impedance of the network is calculated using the first and last node as an ideal electrical input/output contact.

Polarization is considered to be relevant only in the AC regime, so in the DC regime, impedance reduces to simple resistance. Furthermore, with increasing values of the applied voltage, deviations from linearity are commonly observed in macromolecule-based nanodevices [27,28,29,35,36,37]. This has been described as being due to a mechanism of sequential tunneling between the network nodes [35,36,37]. Thus, the resistivity is made dependent on the applied bias as follows:

(3) $$\rho_{l,m}\left(V\right)=\left\{\begin{array}{c} \rho_{MAX},\;{\;eV}_{l,m\;}<\Phi \\ \rho_{MAX}\left(\frac{\Phi }{{eV}_{l,m\;}}\right)+\rho_{min}\left(1-\frac{\Phi }{{eV}_{l,m\;}}\right),\;eV_{l,m\;}\geq \Phi \end{array}\right.$$

where $V_{l,m}$ is the potential drop across the couple of nodes (*l*,*m*), e is the elementary electric charge, and Φ is the potential barrier to be overcome for the transfer of electric charge to take place. We assume as a reference value Φ = 0.22 eV [35,36,37].

The asymptotic values, $\rho_{MAX}$ and $\rho_{min}$, may be tuned on experimental data (still not present here) as in [36], and a stochastic procedure is implemented that assigns the final resistivity value according to the probability:

Pl,m={(4a)exp−βdl,mΦ−eVl,m2,  eVl,m <Φ(4b)exp−βdl,mΦ322eVl,m,  eVl,m ≥Φ

where $\beta =\frac{2\sqrt{2\mu }}{\hbar }$ and μ is the electron mass. The process is not deterministic and the final current oscillates, for each value of bias, around a mean value with fluctuations that testify the coexistence of both tunneling probabilities of Equation (4). As the applied potential increases, the probability described in Equation (4b) becomes higher and the fluctuations smooth out.

- Impedance:

Using Equations (1) and (2), each link is equipped with the elementary impedance:

(5) $$Z_{l,m}=\frac{\rho_{l,m}}{1+{i\varepsilon }_{l,m}\rho_{l,m}\omega }\left[\frac{4d_{l,m}}{\pi \left(D^{2}-{d_{l,m}}^{2}\right)}\right]$$

where $i=\sqrt{-1}$ is the imaginary unit and ω is the angular frequency of the applied voltage. Finally, the network impedance can be calculated by means of a standard numerical procedure [27,28,29] based on the solution of Kirchhoff’s node equations.

This kind of impedance response partially reproduces a typical measurement performed using electrochemical impedance spectroscopy (EIS) [38], a technique that explores the electric and dielectric response of an electrochemical cell when it is tested with a small perturbation at equilibrium [38]. The processes occurring in the electrochemical cell are multiple and involve, for example, diffusion, chemical equilibria, and electron exchange between solid electrodes and dissolved species. A macroscopic description of these phenomena is performed by means of the Randles cell [39,40], i.e., an electrical circuit whose elements are associated with a specific process. In particular, the main element of the Randles cell is the Voigt element, which describes both the electronic conduction (of the electrode functionalized with the appropriate sample and of the electrode–solution interface) and the dielectric properties of the electrode [39]. 

The Voigt element is an RC^α^ parallel circuit, with α = 1 representing an ideal case with a single characteristic time, τ = RC, while α < 1 signals that the sample is not responding as a single object but rather as a set of multiple domains [29,39]. The graphical representation of the Voigt element is a Nyquist plot, i.e., the plots of the imaginary vs. the real part of the impedance associated with the RC^α^ circuit.

Other elements may be added to the Randles cell, as, for example, the low-frequency Warburg impedance or the high-frequency resistance solution [38,39,40], although both these elements do not give insights about the sensing action, which is usually limited to the analysis of the responses associated with the Voigt element. 

For this reason, we expect that by subtracting the baseline, associated with elements of the cell that do not change in the measurements, the answer is entirely driven by Equation (5).

### 3.2. Materials

The structures compared here concern the free aptamer TBA in its G-quadruplex form in the presence of different cations (Table 1). 

Although the ability of TBA to adapt its conformation to the presence of other cations such as Ba^2+^ and NH_4_^+^ is known and the use of this aptamer to detect these cations has been studied [26], at the moment, there are no data regarding these structures.

Therefore, we focused on TBA stabilized in the presence of Pb^2+^, Sr^2+^, Mn^2+^, and K^+^ in the stoichiometry 2:1 (2 cations:1 aptamer) and 1:1.

The 3D structures were taken from the Public Data Bank [11] and are shown in Table 1.

Some of these PDB entries refer to the same structure, although measured/calculated in different specific conditions: in particular, 1c34, 1c38, and 148d for TBA in the presence of K^+^, stoichiometry (1:1); 1c32 and 1c35 for TBA in the presence of K^+^, stoichiometry (2:1); 1qdh and 1qdf for TBA in the presence of Mn^2+^; and 7d31 and 7d32 in the presence of Pb^2+^. 7d33 is a mutant of 7d31 (G8C). 

After some preliminary analyses, we conclude that the methods given here are unable to resolve significant differences within each group, and for the subsequent analyses contained in this article, only one of them was used, specifically 1c34 for K^+^, 1c32 for K^+^ 2:1, 1qdh for Mn^2+^, and 7d31 for Pb^2+^.

## 4. Conclusions

In this article, we investigate the effects of cations, some of which are of particular interest for human health, on the structural and electrical characteristics of the anti-thrombin TBA aptamer. This aptamer is the subject of various studies not only for its clinical applications but also for its ability to modify its structure in the presence of different cations, so that it can be used for sensory purposes. 

Its specific 3D spatial organization, i.e., a G-quadruplex structure, is observed in the presence of all the analyzed cations, although small but significant differences between the structures appear. These differences are expected to produce different electrical responses when the aptamer is used to sense a specific cation. Specifically, we calculate, for a set of deposited structures of TBA, the predicted impedance from electrochemical measurements or the observable conductance at a metal–aptamer–metal junction. In conclusion, it has been calculated that the presence of lead or strontium produces much more pronounced responses than those produced by potassium, which is a good indication to produce highly selective sensors.

## Figures and Tables

**Table 1:** The analyzed structures of the aptamer TBA.

State	Ligand	Method	PDB#	Ref.
free	K^+^	NMR	148d	[18]
free	Pb^2+^	X-ray	7d31	[9]
free	Pb^2+^	X-ray	7d32	[9]
free	Pb^2+^	X-ray	7d33 *	[9]
free	2K^+^	NMR	1c32	[21]
free	2K^+^	NMR	1c35	[21]
free	K^+^	NMR	1c34	[21]
free	K^+^	NMR	1c38	[21]
free	Sr^2+^	NMR	1rde	[22]
free	2Mn^2+^	NMR	1qdf	[19]
free	2Mn^2+^	NMR	1qdh	[19]
complexed	K^+^-thrombin	X-ray	4dii	[23]

* mutant structure G8C.

## Data Availability

The data presented in this study are available on request from the corresponding author.
